# A Rare Case of Cutaneous Plasmacytosis in a Korean Male

**DOI:** 10.1155/2017/3032941

**Published:** 2017-08-07

**Authors:** Corey Georgesen, Meenal Kheterpal, Melissa Pulitzer

**Affiliations:** ^1^Department of Dermatology, NewYork-Presbyterian Hospital/Weill Cornell Medical Center, New York, NY, USA; ^2^Department of Medicine, Memorial Sloan Kettering Cancer Center, New York, NY, USA; ^3^Department of Pathology, Memorial Sloan Kettering Cancer Center, New York, NY, USA

## Abstract

Cutaneous and systemic plasmacytosis are reactive disease processes that occur in middle-aged Japanese and Chinese men. Systemic plasmacytosis, defined by plasmacytic infiltration of two organ systems, might rarely progress to lymphoma. Cutaneous plasmacytosis, however, is chronic and benign and is characterized by the development of multiple plasma cell-rich infiltrates in the skin. We present a case of cutaneous plasmacytosis in a 46-year-old Korean male. The patient demonstrated classic features of the disease entity, including disseminated red-brown plaques, differentiated plasmacytoid infiltrates on biopsy, hypergammaglobulinemia, and the absence of systemic disease.

## 1. Introduction

Cutaneous and systemic plasmacytosis, originally considered variants of Castleman's disease, are thought to be reactive processes that occur largely in Asian males who are between 20 and 55 years old [1]. Systemic plasmacytosis was originally described by Watanabe et al. [2] in 1986 who reported two patients with multiple pigmented skin lesions, generalized lymphadenopathy, and polyclonal hypergammaglobulinemia. Systemic plasmacytosis, defined by involvement of two organ systems (i.e., lymph nodes, liver, spleen, or lung), can rarely progress to lymphoma.

Cutaneous plasmacytosis is characterized by a chronic and benign course [3]. The entity was described by Yashiro [4] in 1976, prior to the Watanabe systemic variant. Kitamura et al. [5] first coined the term “cutaneous plasmacytosis” to refer to the proliferation of mature plasma cells in the skin, resulting in multiple cutaneous plaques with polyclonal hyper-*γ*-globulinemia. Cutaneous plasmacytosis is now a well-recognized (albeit rare) entity, and physicians should be aware of diagnostic workup algorithms and surveillance monitoring recommendations to rule out systemic progression.

The distinction between cutaneous and systemic plasmacytosis is often ambiguous. Some cases of cutaneous plasmacytosis have been described as harboring occult areas of extracutaneous involvement, for example, within lymph nodes and bone marrow [3]. This has even led some authors to favor the nomenclature “cutaneous and systemic plasmacytosis” [6] to describe all cases. Herein, we describe a case of cutaneous and systemic plasmacytosis diagnosed in a Korean patient and review the literature in this population.

## 2. Case Presentation

A 46-year-old male Korean patient presented to the referring dermatologist with a ten-month history of a nonpruritic, nontender, and insidious rash on the face, neck, and trunk. The patient had no prior medical history and did not take any medications. He specifically denied fevers, chills, weight loss, lymphadenopathy, dyspnea, abdominal pain, and changes in urination. He had not attempted any treatment for the rash prior to the visit.

**Figure 1:**
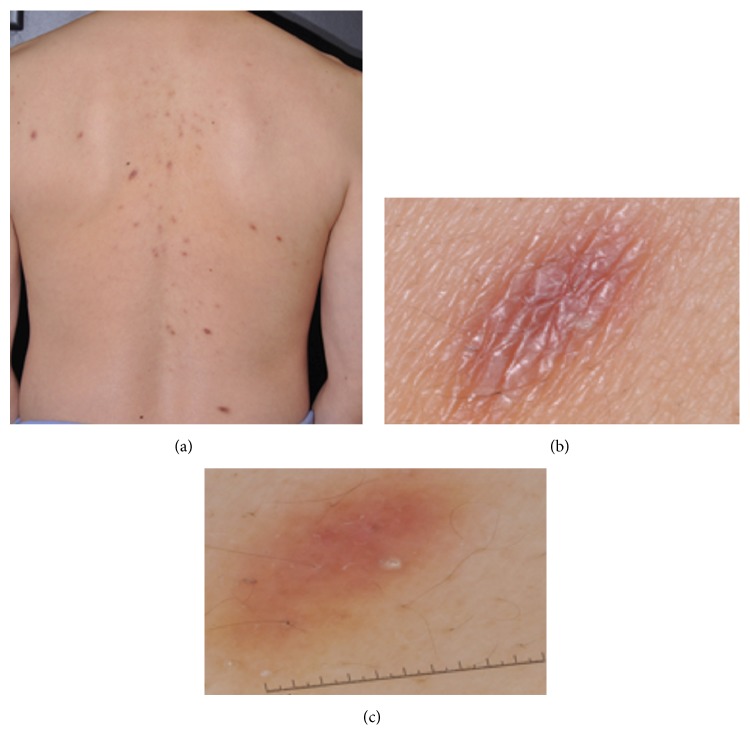
(a) Numerous polygonal-oval red-brown plaques with well-demarcated borders in a symmetric distribution on the back. (b) Close-up view shows thickened epidermis with coarse wrinkling, mild scale, and underlying elongated region of erythematous to tan discoloration. (c) Dermoscopy reveals slight pink hue and prominence of network.

Physical examination revealed numerous disseminated well-demarcated red-brown nonscaly plaques on the patient's face, neck, chest, and back. Plaques ranged from 0.5 cm to 2 cm in size and were distributed haphazardly (Figures 1(a) and 1(b)). Dermoscopic exam was overall nondiagnostic and remarkable for a slight pink hue and prominence of network (Figure 1(c)). Laboratory evaluations of complete blood count and metabolic panel were normal. HIV, RPR, hepatitis serologies, and QuantiFERON Gold were negative. A skin biopsy was performed, and the patient was referred to our clinic for further care.

**Figure 2:**
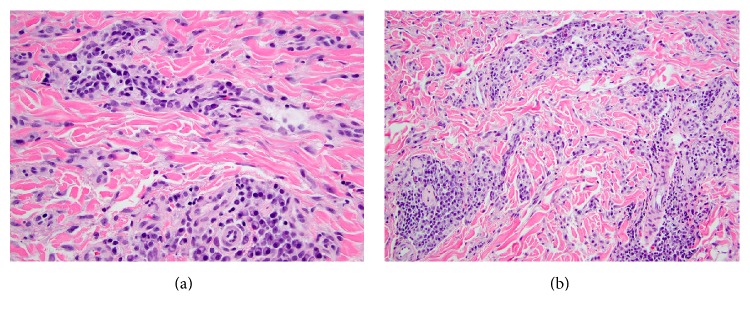
(a) Hematoxylin and eosin, 10x. Superficial and deep perivascular small round blue cell infiltrate below a moderately hyperplastic epidermis with elongated rete ridges showing basal layer hyperpigmentation. (b) Hematoxylin and eosin, 40x. Perivascular infiltrates are comprised of plasma cells and fewer small lymphocytes.

**Figure 3:**
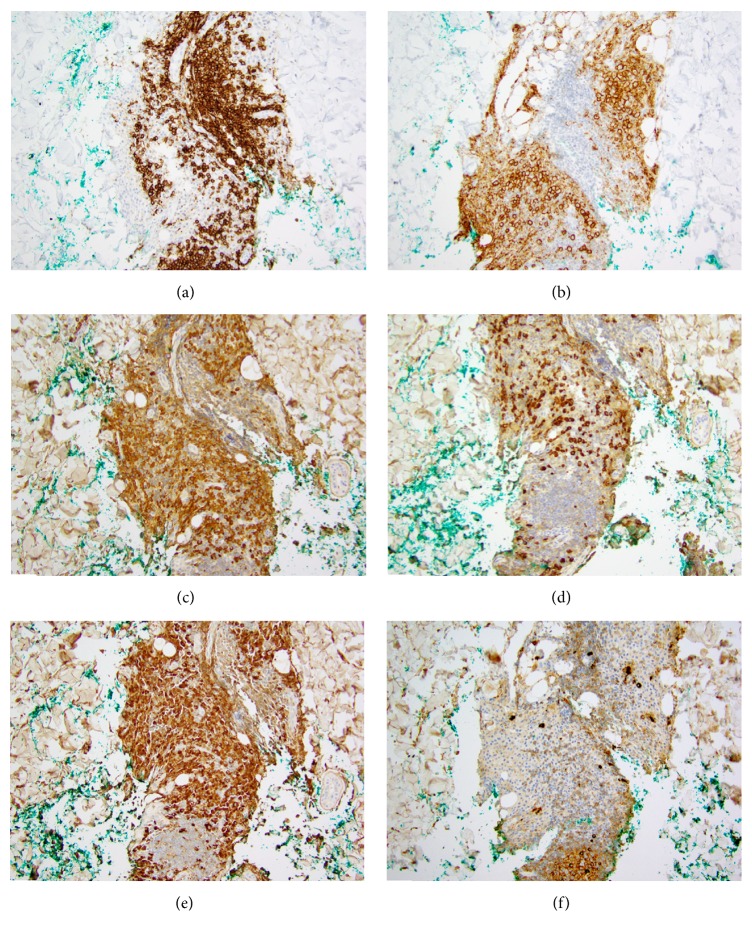
(a) CD20 highlights a few aggregates of small B-lymphocytes. (b) CD138 shows the relative distribution of plasma cells around B-cell aggregates. (c) Kappa and (d) lambda immunohistochemistry shows a 3-4 : 1 ratio. (e) IgG is diffusely present in these plasma cells, while (f) IgM is mostly absent.

Histopathology revealed prominent superficial and deep perivascular and periadnexal infiltrate of hyperchromatic mononuclear cells (Figure 2(a)), with focal localization to small nerve twigs. Higher power examination revealed predominance of plasma cells lacking atypia in a background of small lymphocytes (Figure 2(b)). Immunohistochemical stains highlighted the aggregates of CD20+, CD79A+, and B-lymphocytes (Figure 3(a)) amidst more numerous CD138+ (Figure 3(b)), IgD+, IgG+, and IgM− plasma cells (Figures 3(c) and 3(d)). Kappa and lambda in situ hybridization labeled the plasma cell population with a polytypic pattern including slight kappa predominance (normal finding) (Figures 3(e) and 3(f)). The findings were compatible with cutaneous plasmacytosis.

A peripheral blood analysis identified a polyclonal gammopathy with elevated IgG; however, there was no indication of any associated lymphoproliferative process. Positron emission tomography (PET) and CT scans showed prominent bilateral inguinal lymph nodes measuring 1.5 centimeters. Interleukin-6 was elevated at 11.8 picograms per milliliter.

The patient was offered a lymph node biopsy of his inguinal lymph nodes, which he deferred. He is currently being followed up by his primary provider and is applying topical clobetasol in weekend pulse dosing, with improvement of most skin lesions and without any evidence of systemic progression.

## 3. Discussion

Plasmacytosis is a rare entity. In the most recent review by Wagner et al. [3], an estimated 67 cases had been previously reported. Almost all the reported patients are Japanese. A few cases of Chinese, Thai, and Caucasian patients have occurred [6, 11–14]. To our knowledge, cutaneous and systemic plasmacytosis has been reported in only four Korean patients [7–10] (Table 1).

Clinically, patients present with ovoid, poorly demarcated, red-brown patches and plaques, typically without scale, crust, or any other surface change. The trunk is most commonly affected, with relative sparing of the extremities. The course of cutaneous disease is chronic, with a tendency for recurrence. Reported treatments include topical and systemic corticosteroids, topical tacrolimus, antibiotics, psoralen with ultraviolet A, and chemotherapy [6, 11]. Extracutaneously, lymphadenopathy (detected either clinically or radiographically) is the most common associated finding. Other symptoms tend to be nonspecific, including fever, chills, weight loss, and fatigue [3, 6, 11]. Systemic involvement has rarely been reported to manifest with hepatosplenomegaly, interstitial pneumonitis, and glomerulonephritis [3]. Physicians should therefore screen for involvement of these organs on initial workup.

Histopathologically, plasmacytosis is classically characterized by a dense, superficial, and deep perivascular and periadnexal infiltrate of mature plasma cells. These plasma cells are typically polyclonal and exhibit little to no atypia [1, 15]. Perineural involvement, and even infiltration of the nerve fascicles, is common [16]. The epidermis is often acanthotic with basal layer hyperpigmentation [1, 3]. Additionally, authors have reported that reactive germinal centers may occasionally be found [1]. The histopathologic differential diagnosis includes monoclonal hematolymphoid malignancies such as marginal zone lymphoma, cutaneous plasmacytoma, and leukemia cutis. Other considerations include polyclonal plasma cell-rich infiltrates such as B-cell pseudolymphomas, multicentric Castleman's disease, borreliosis, and syphilis [1, 3, 6, 11, 15]. These entities can often be identified by attention to a combination of clinical, serologic, and molecular studies for B-cell clonality (Table 2).

It is not clear that all patients with cutaneous plasmacytosis require a lymph node biopsy. However, recognizing a lack of consensus on that matter, any patient with findings on PET scan, lymphadenopathy, or other positive findings on a review of systems should be investigated further. Cases have been described in which patients were found to have plasma cell infiltrates within lymph node biopsies despite lack of radiographic evidence of systemic disease [17, 18]. Our patient was offered a lymph node biopsy given his borderline enlargement of inguinal lymph nodes but he deferred it given negative review of systems after a thorough review of the concomitant risks and benefits. To date, he has not experienced other disease manifestations.

While the relationship of plasmacytosis and overt lymphoma has been described, the overall incidence and implicated pathogenesis are unclear. Previous investigations have not been able to discern whether plasmacytosis is in fact a reactive or neoplastic process. One hypothesis is that plasmacytosis is a variant of Castleman's disease, and in fact both entities often exhibit elevated interleukin-6 levels (as did our case patient), although HHV-8 is reported to be negative in plasmacytosis patients [19, 20]. Other hypotheses center around genetic factors, environmental influence, and infectious triggers [3]. It is worth noting that our patient had spent most of his life in the United States, thereby suggesting that genetic (more than environmental) factors are implicated in the disease pathogenesis.

This case highlights clinical presentation, diagnostic workup, and histopathology of cutaneous plasmacytosis. Despite originally being described solely in Japanese patients, this entity can occur in other Asian populations. To our knowledge, this is the first reported case of isolated cutaneous plasmacytosis without systemic involvement in a Korean patient living in North America.

Histopathologically, when dense plasma cells infiltrates are observed, both monoclonal and polyclonal plasma cell-rich neoplastic and inflammatory processes must be excluded prior to the diagnosis of plasmacytosis (Table 2). While “cutaneous plasmacytosis” may harbor occult plasma cell infiltrates within extracutaneous organs, it seems that systemic involvement may not bear poor prognosis, although systemic blood analysis for monoclonal gammopathy, CT/PET scan, and regular clinical monitoring at one-month to three-month intervals are recommended.

## Figures and Tables

**Table 1:** Reported cases of plasmacytosis in Korean patients [7–10].

Case number	Author	Age/sex	Duration prior to diagnosis	Extracutaneous involvement	Comorbidities	Treatment/response
(1)	Lee et al. [7]	52/M	4 years	Lymphatics, spleen	None	Melphalan/improved

(2)	Lee et al. [8]	54/M	5 years	Lymphatics, bone marrow, kidney	CKD	Prednisolone, mycophenolate/moderate regression

(3)	Lee et al. [9]	48/F	3 years	Lymphatics, lungs	None	Prednisone, melphalan/mild improvement

(4)	Amin et al. [10]	49/M	4 years	Lymphatics	None	Cyclophosphamide, doxorubicin, vincristine, prednisone and then rituximab/minimal response

F, female; M, male; CKD, chronic kidney disease.

**Table 2:** Differential diagnostic considerations in plasmacytosis.

	Diagnostic entity	Features	Immunohistochemistry/special stains
Neoplastic (monoclonal)	Marginal zone lymphoma	Plasma cells at periphery of germinal centers	CD20, CD79a, PAX5, BCL2+; BCL6, CD10−
	Plasmacytoma	Large infiltrate of monoclonal plasma cells	CD79a+; CD19, CD20−
	Leukemia cutis (plasma cell leukemia)	Infiltrates of atypical-appearing plasmacytoid cells	CD38, CD138+; CD20+/−; CD19, CD49e−

Inflammatory (polyclonal)	Castleman's disease (plasma cell variant)	Lymph nodes with hyperplastic follicles and interfollicular sheets of (lambda restricted) plasma cells	HHV-8+
	Pseudolymphoma	Circumscribed follicles of lymphocytic infiltrates with plasma cells at periphery; clinical correlation is paramount	Directed to rule out lymphoma, which is more likely BCL-6 and CD10+ outside follicle and BCL-2+ within follicle; Ki-67, more diffuse staining in reactive germinal centers than lymphoma

Infectious (polyclonal)	*Borrelia* (erythema migrans, acrodermatitis chronica atrophicans)	Perivascular lymphocytic infiltrate, rich in plasma cells	Wright-Giemsa, Warthin-Starry (silver stains)
	Syphilis	Dense plasma cell predominant dermal infiltrate, elongated rete ridges, and endothelial swelling	Silver stains, *T. Pallidum* immunohistochemistry
